# An adaptive fall-free rehabilitation mechanism for ischemic stroke rat patients

**DOI:** 10.1038/s41598-018-37282-3

**Published:** 2019-01-30

**Authors:** Chi-Chun Chen, Ching-Ping Chang, Chin-Lung Yang

**Affiliations:** 1 Department of Electronic Engineering, National Chin-Yi University of Technology, Taichung, Taiwan; 2 Department of Medical Research, Chi Mei Medical Center, Tainan, Taiwan; 3 Department of Electrical Engineering, National Cheng Kung University, Tainan, Taiwan

## Abstract

Today’s commercial forced exercise platforms had been validated not as a well-designed rehabilitation environment for rats with a stroke, for the reason that rat with a stroke cannot take exercise at a constant intensity for a long period of time. In light of this, this work presented an adaptive, fall-free ischemic stroke rehabilitation mechanism in an animal model, which was implemented in an infrared-sensing adaptive feedback control running wheel (IAFCRW) platform. Consequently, rats with a stroke can be safely rehabilitated all the time, and particularly at full capacity for approximately one third of a training duration, in a completely fall-free environment according to individual physical differences by repeated use of an acceleration/deceleration mechanism. The performance of this platform was assessed using an animal ischemic stroke model. The IAFCRW therapy regimen was validated to outperform a treadmill and a conventional running wheel counterpart with respect to the reduction in the neurobehavioral deficits caused by middle cerebral artery occlusion (MCAo). IAFCRW is the first adaptive forced exercise training platform short of electrical stimulation-assistance in the literature, and ischemic stroke rats benefit more in terms of the behavioral tests run at the end of a 3-week rehabilitation program after a stroke thereby.

## Introduction

Medical expense in ischemic stroke patents has long been considered a huge burden to health care companies in many countries, and patients often experience difficulty performing their activities of daily living^1,2^. Therefore, an effective and completely safe rehabilitation program is seen as crucial to improve patients’ quality of life, and increasing evidence has suggested that physical exercise can enhance the neurological function and motor recovery after stroke^3–5^. In addition, post-ischemic stroke exercise rehabilitation has been proposed as a practical cerebral stroke treatment^6–8^. Rodent, e.g. rat, injury models are frequently employed as a preliminary approach to validating the effectiveness of physical rehabilitation methods^5,9,10^, and rats are trained at a fixed running speed over a specified time period. These rehabilitation methods have been validated as effective in cerebral stroke prevention^11,12^, while has been found not to be as effective in cerebral stroke rehabilitation^13,14^ as in prevention. A reason behind this is that these forced platforms are designed to train normal and healthy rats, but their training parameters could not be directly applied to cerebral stroke rehabilitation programs.

There exist a number of limitations on today’s training platforms, including treadmills and running wheels. Rats are stimulated when they run at the end of treadmill runways, accounting for part of a physiological outcome^15^. Therefore, treadmill data were very likely to be collected with an interference factor induced by electric shocks. Moreover, electric shock may impose stress on or directly hurt the rats during rehabilitation^13,14^. Running wheel platforms can be designed as either a voluntary or a motorized form. As its name indicates, rats are permitted to run voluntarily on a voluntary running wheel. However, due to individual differences, this type of running wheel platforms often yields large variations in the final results. To avoid such discrepancies, rats often needed to be selected carefully in advance^16,17^, and voluntary running wheels were not treated as a key issue. As pointed out in^18^, rats were afraid of running, held on to the cross bars of a wheel or even stopped running, when trained using commercially available motorized running wheels (MRWs). In addition, commercially available running wheels with a diameter of 35 cm and a width of 12 cm were generally too small for average sized white rats, and were liable to cause rat patient injury^19^. In addition, it is more difficult to run on a curved runway than on a flat one, and rats often accidentally fell or tumble^20,21^. Therefore, this study aims to develop and then integrate an adaptive training mechanism into a larger sized running wheel for an improved motor function recovery after stroke.

For the sake of an improved recovery quality, a training mechanism must be made adaptive together with a low level of interference according to the physical conditions of rats during rehabilitation. Previous studies suggested that the stress response to electrical shocks in treadmills resulted in adverse physiological injuries, such as adrenal hypertrophy, splenic atrophy and circulating corticosterone^22–24^. For those taking a rehabilitation program, stress response could be a destructive factor to their recovery^13^, and is as well an uncontrolled parameter that can affect the final neurological outcomes. Therefore, it is advantageous to remove such potential disadvantages for the sake of clinical research. This study reviewed a number of running wheel platforms that were developed to assist in the effective recovery of rats with an ischemic stroke but short of electric shock. Utilizing an IR sensor-embedded wheel module to detect the running position of a rat, an acceleration/deceleration mechanism was enabled herein in such a way that rats were rehabilitated well in a completely fall-free environment, and adaptively within their capacity for an improved motor function recovery and a reduced cerebral infarct volume.

## Methods

Development of the infrared-sensing adaptive feedback control running wheel (IAFCRW) is detailed as follows. As an IR sensor-equipped measurement apparatus, IAFCRW was designed to sense the running positions of rats and to adaptively adjust the exercise speed. An acceleration/deceleration mechanism was built, and was controlled by a micro-processor for stroke rat rehabilitation according to individual physical status. Then, the effectiveness of this platform was verified with an animal ischemic stroke model. One week after middle cerebral arterial occlusion (MCAo) surgery, the animals were assigned to three experimental groups, i.e. treadmill, MRW and IAFCRW groups, to participate in a 3-week rehabilitation program. Finally, an inclined plane, a beam walking test and MCAo were used to evaluate their recovery outcomes.

### Infrared-sensing adaptive feedback control running wheel system

**Figure 1:**
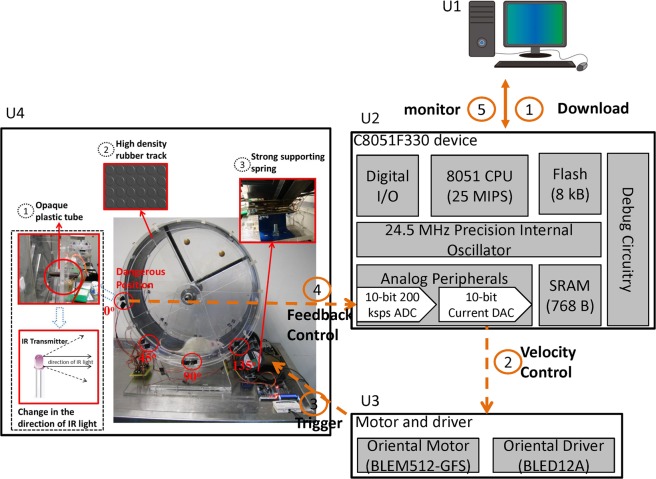
System architecture of the presented infrared-sensing adaptive running wheel rehabilitation platform. Stereogram of the platform together with key features.

A novel infrared-sensing adaptive feedback control running wheel system was presented herein as an extension of a piece of prior studies for an exercise quantification of animals, such as rats^19^, and offered a fully automated adaptive and fall-free rehabilitation environment to rat patients. As illustrated in Fig. 1, it involves a computer (U1), a microcontroller (MCU) (U2), a motor and a driver (U3) and an infrared sensing running wheel platform (U4).

U1 downloads a rehabilitation training software program to U2 through an RS-232 serial adapter. U2 is a C8051F330 development board, using pulse-width modulation (PWM) to initiate a 10-bit digital-to-analog converter (DAC) and to control the motor driver (BLED12A, Oriental Driver, Japan). The motor (BLEM512-GFS, Oriental Motor, Japan) drives the rotation of U4. After U4 has been initiated, infrared sensors are activated to monitor the position of a rat.

The interior of the wheel can be divided into the safe and the unsafe zones. Ideally, rats could always catch up with the running wheel, and stayed in the safe zone during rehabilitation. In practice, rats were occasionally brought up to the unsafe zone, lost the support from the railway, and fell down consequently once unable to catch up. As soon as the lower part of a rat’s body reached the 0 degree position in Fig. 1, a deceleration mechanism was automatically enabled by U2 as a preventive measure in IAFCRW, the rat were brought back to the safe zone, an acceleration mechanism was enabled right away, and the wheel continued to accelerate again for uninterrupted rehabilitation. For comparison purposes, pairs of infrared sensors were positioned in the same way as in an MRW counterpart in the absence of any deceleration mechanism.

**Figure 2:**
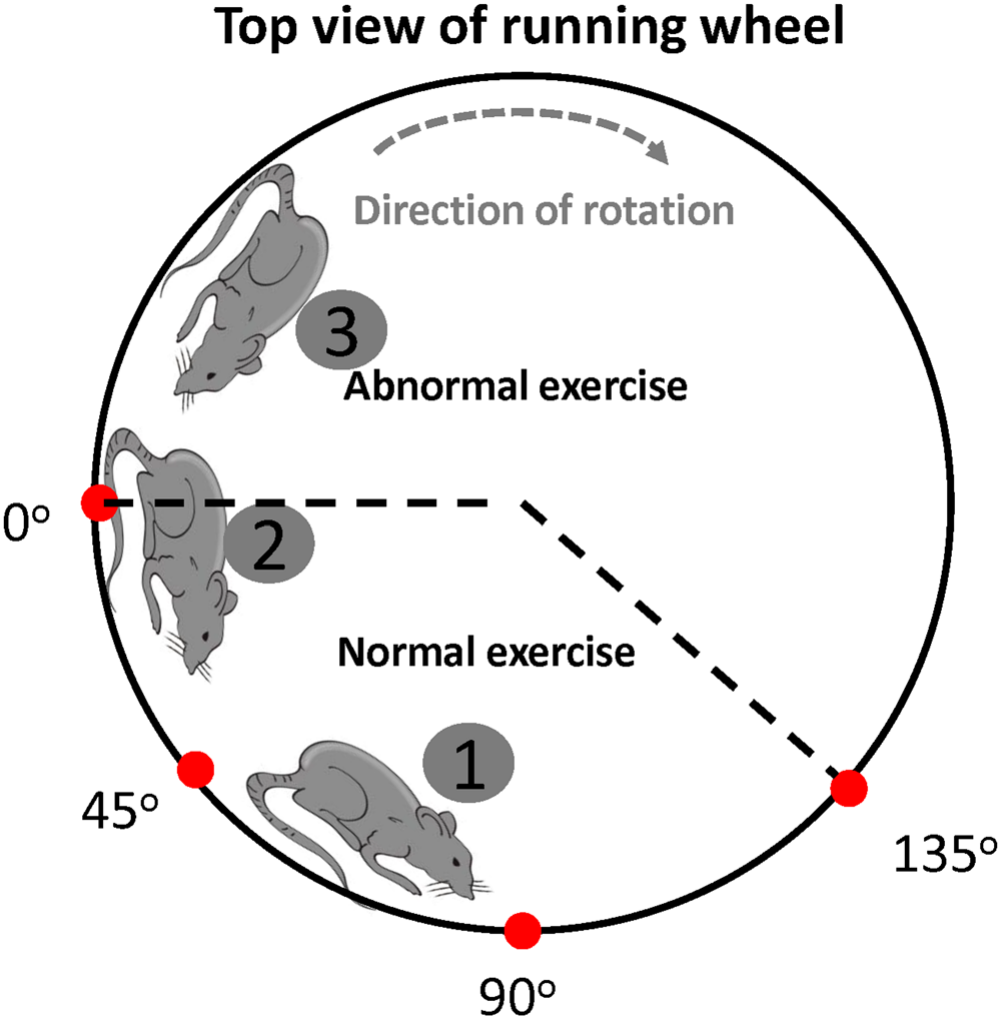
The interior of a running wheel. Spot 2 is the threshold between the safe and the unsafe zones. Once beyond spot 2, a rat will lose its support from the railway, and fall consequently.

As illustrated in Fig. 1, U4 is mechanical drawing of the presented apparatus built in the Chimei Hospital Laboratory. The running wheel mechanism is actually a modified version of the previously developed ISRW^19^, but with 3 key features illustrated in the stereogram of Fig. 1. The first key feature was an improved sensitivity of IR position sensing, the second was a rat friendly rehabilitation environment using a high-density rubber track, and the third was an improved driving motor mounting technique using a strong spring embedded into an L-shaped supporting iron frame on the side, as shown in Fig. 1 (U4). The infrared transmitter and sensor pair at the 0 degree position were tubed to reduce the scattering range, and accordingly the IR sensor was trigger with an improved sensitivity once the 0 degree position was reached by the lower part of a rat’s body. Four pairs of transmitters and sensors were equally mounted between 0 to 135 degrees, an area defined as the safe zone^19^, and the received IR signals were delivered to an MCU through general-purpose input/output port 1 (GPIO P1). The area beyond the 0 degree position was defined as the unsafe zone, and hence the 0 degree position was the threshold between both zones. As illustrated in Fig. 2, the interior of the IARCRW can be divided into the safe exercise zone, marked by spots 1 and 2, and the unsafe exercise zone, marked by spot 3. Once the IR sensor at the 0 degree position was triggered by the lower part of a rat’s body, the deceleration mechanism was enabled instantly, and the rat was brought back to spot 1 safely for persistent rehabilitation, meaning that the fall-free requirement was fulfilled. In contrast, rehabilitation on MRW inevitably resulted in injury to rats, once they were brought up to spot 2 or even 3, since MRW was not equipped with a position detection and deceleration mechanism. Hence, it is concluded that the IAFCRW platform was experimentally validated as a fall-free and definitely a much safer rehabilitation environment than the MRW counterpart.

### Automated adaptive acceleration and deceleration training models

For rats with a stroke, persistent forced exercise for a long period of time at a fixed speed leads to less therapeutic effects than voluntary exercise^13,14^. Therefore, an adaptive rehabilitation mechanism is required to meet the individual physical requirements. An automated training model was constructed by the curve fitting of manual training data on rats. This model not only reduces human error in operations, but covers as much physical diversity as possible for injured rats. It must be noted that a formal rehabilitation training program does not apply to the rats for the construction of the adaptive training model.

### Automated adaptive acceleration training model

**Figure 3:**
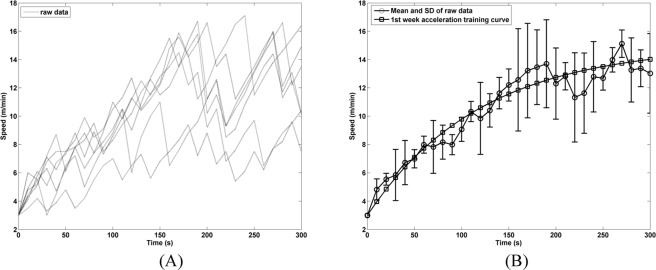
Adaptive acceleration curve construction. (**A**) The dotted curve represents the raw data on day 3 of a manual rehabilitation training program one week after MCAo surgery. (**B**) The curve, marked by squares, is the adaptive acceleration training curve best fitted to the averaged raw data, marked by circles, for week 1.

Rehabilitation began one week after an MCAo surgery. During the first three days of the rehabilitation program on the IAFCRW platform, rats were trained in a manual manner to get the raw data for model construction, were assured to get familiar with the training environment at the onset, and the training intensity that each rat could withstand was measured as well. In the manual mode, the running wheel was gradually accelerated. If a rat was not able to keep pace with the running wheel, then the speed was turned down as a way to prevent the rats from being overwhelmed by the exercise and to complete their rehabilitation. A family of dotted curves in Fig. 3(A) represented the raw data of a group of seven rats on day 3, and a formal rehabilitation training involved another group of 30. Each rat achieved different ultimate speed, although a similar exponential growth in speed was shared. A close observation on Fig. 3(A) reveals that 5 out of 7 rats reached a speed beyond 15 m/min during the rehabilitation, but not for a long period of time, whereas the remaining two failed, and were ruled out in the subsequent curve fitting accordingly. As the first step, the average performance of the 5 rats, marked by circles, was presented in Fig. 3(B) with standard deviation error bars, and the curve fitting was then performed. The other curve, marked by squares therein represents the best fit of the acceleration model in Eq. (1), and the values of the best fit parameters A, B and τ in Eq. (1) were found to be 12, 3 and 120, respectively. Particularly, a good model fit is indicated by an R-squared value of 0.93. The speed curve for week 1 was slightly shifted upward as the week 2 case so as to enhance the exercise intensity, but with the initial and the ultimate speeds fixed. Consequently, the *τ* value of the curve, marked by triangles, was tuned to 100. Weeks 2–3 shared the same rising slope at t = 0, although the week 3 case had a stretch training period of 60 min.

1 $$V=A\times (1-{e}^{-t/\tau })+B$$

### Automated adaptive deceleration training model

**Figure 4:**
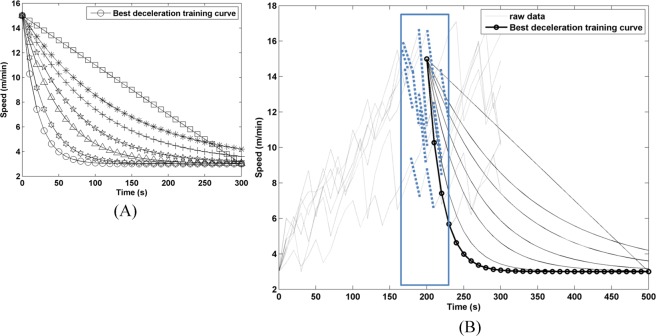
A family of deceleration curves. (**A**) A family of deceleration training curves for curve fitting, (**B**) superposition of the best fit curve in (**A**) onto the raw data in Fig. 3.

As a fall prevention mechanism, a deceleration training model is far more important than an acceleration one. A group of seven rats were involved to construct an adaptive deceleration training mode. Beginning one week after the MCAo surgery, a 3-day test was performed to construct the adaptive deceleration model. A family of deceleration curves in Fig. 4(A) were treated as candidates to best describe the deceleration characteristics of rats after reaching the danger zone. First, a linear deceleration model, marked by squares, was described by Eq. (2) with the parameters *A* and *B* set to 375 and 25, respectively, and gives a deceleration of 2.4 m/s^2^ together with an R-squared value of 0.84. The other curves were tested by turns leftward, and the leftmost curve, marked by circles, was found to best fit the raw deceleration data. The best fit exponential decay model was described by Eq. (3) with *A*, *B* and *τ* set to 12, 3 and 20, respectively, and gives an R-squared value of 0.88. The wheel decelerated rapidly, but not abruptly, to below 6 m/min within 30 s, for safety concern of rats, as a way to avoid falling forward. Superimposed on Fig. 4(B) for comparison purposes, the best fit curve gives an average deceleration of 18.75 m/s^2^ over the time interval (0, 30) using Eq. (4), while substitution of the raw deceleration data between 170 and 230 s, highlighted in dotted blue, into Eq. (5) gives an average deceleration of 18.44 m/s^2^. There is a good agreement between the manual and the best fitted average decelerations, that is, an error as low as 1.6% is found using Eq. (6). In other words, this accounts for a superior R-squared value in the best fit exponential decay model than the linear one.

2 $$V(t)=(A-t)/B$$

3 $$V(t)=A\times ({e}^{-t/\tau })+B$$

4 $${m}_{{automated}}=V^{\prime} (t)=A\times ({e}^{-t/\tau })\times (\,-\,t\,/\,\tau )$$

5 $${m}_{{manual}}=\frac{V({t}_{1})-V({t}_{2})}{{t}_{1}-{t}_{2}}$$

6 $${m}_{{error}}=\frac{{m}_{{automated}}-{m}_{{manual}}}{{m}_{{automated}}}\times 100 \% $$

### Software

**Figure 5:**
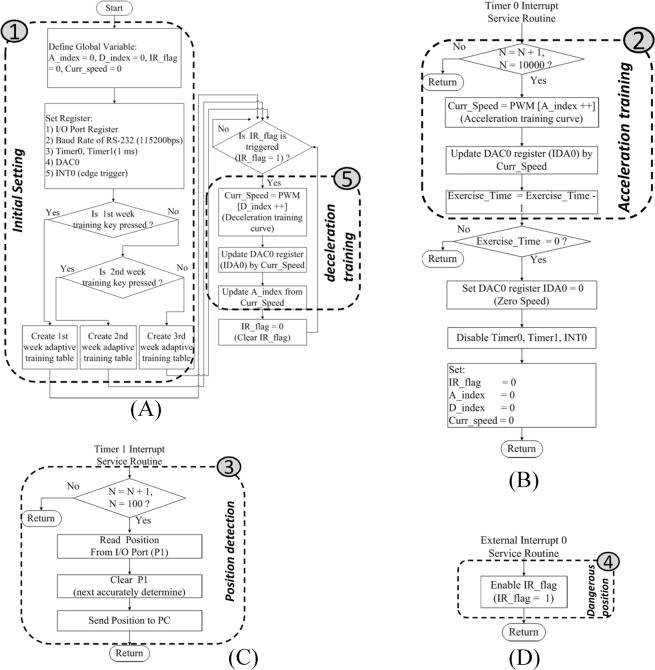
A software program on a microcontroller. (**A**) Main program flow of a microcontroller. (**B**) Flowchart of timer 0 service routines of the microcontroller. (**C**) Flowchart of timer 1 service routines of the microcontroller. (**D**) Flowchart of external interrupt 0 service routine of the microcontroller.

The presented fall-free rehabilitation mechanism is implemented as a software program running on a microcontroller. The main program flow is shown in Fig. 5(A), and the three interrupt service routine processes are shown in Fig. 5(B–D). The main program was responsible for system initialization, danger zone identification and adaptive deceleration model initiation. The three interrupted service routine programs comprised adaptive acceleration model initiation, position detection and danger zone signal triggering. The operational architecture of the entire program could be divided into five parts. Part 1 consists of the initial establishment of the global variables and internal microcontroller registers. These registers include input and output (I/O port) pins, a basic time interval interrupt, an external interrupt trigger, RS-232 transmission rate (115,200 bps) and a DAC (Fig. 4(A)). Part 2 initializes the acceleration training model (Fig. 5(B)), using the Timer 0 interrupt service routine. Part 3 reads and records four sets of infrared sensing signals, using the Timer 1 interrupt service routine (Fig. 5(C)). These signals are connected to the P1 register I/O port. Real-time monitoring on rat positions during the trial can be read from the P1 register (Fig. 5(C)). Part 4 detects the danger zone, i.e. the 0 degree position, using an external interrupt service routine. When a rat reaches the danger zone, this interrupt is triggered to set up a danger zone flag (IR_flag = 1) (Fig. 5(D)). Part 5 determines whether the danger zone flag has been set. Once set, the deceleration training model will be enabled instantly to slow down the running wheel for rat protection (Fig. 5(A)).

### Experimental animals

The experimental animals, weighing approximately between 270–320 g and provided by the National Laboratory Animal Center, Taiwan, were Sprague-Dawley male rats. A group of seven rats were involved to construct an adaptive deceleration training mode, and another group of 30 got involved in a rehabilitation program. The rats were maintained in an air conditioned animal chamber, and the chamber temperature was set at 24 ± 1 °C. A light/dark cycle of 12 hours with lights turned on at 6 am and off at 6 pm was used, and unlimited water and feed were provided. All of the experiments were conducted in the daytime condition under light. The experimental procedures were approved by the animal ethics committee of Chi Mei Medical Center, Ministry of Science and Technology, Taiwan. All methods were performed in accordance with the approved guidelines and regulations.

### Experimental groups and exercise training

**Figure 6:**
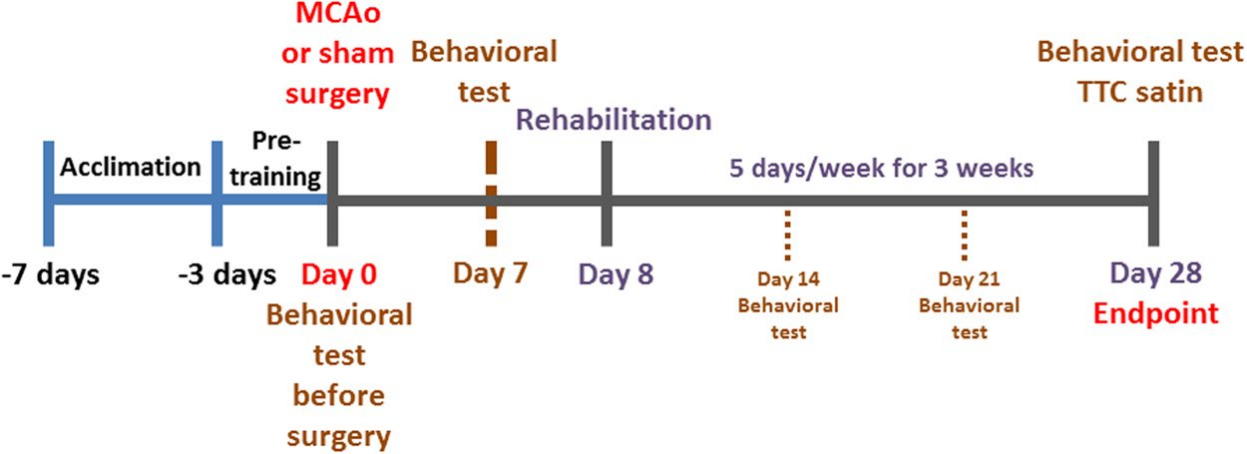
Experimental timeline of the rehabilitation process for ischemic stroke to compare the performance among the treadmill, MRW and IAFCRW platforms.

First, the rats were randomly divided into the following five groups: IAFCRW (n = 10), treadmill (n = 10), MRW (n = 10), sham (n = 10), and control (n = 10) groups. MCAo surgery was performed for all of the groups except the sham group, and exercise rehabilitation training was not provided for the sham and the control groups. The exercise training groups (IAFCRW, treadmill and MRW) took a three-week rehabilitation training program one week after a surgically triggered stroke. During weeks 1–2, the treadmill and MRW groups received a 30 min training at a speed of 20 m/min five days per week. During week 3, the training duration was extended to 60 min but at the same speed^25,26^. However, the IAFCRW group received exercise training using the acceleration and deceleration models developed in this study. Thus, the treadmill and MRW groups took fixed-speed rehabilitation training, whereas the IAFCRW group had no choice but to receive a time-varying speed counterpart during the rehabilitation exercise. As illustrated in Fig. 6, the day that MCAo or Sham surgeries were performed was viewed as a point of reference, represented as Day 0, and all the motor function assessments, including mNSS, incline plane and beam balance tests, were conducted a day before and on a weekly basis after the surgeries, that is, Days -1, 7, 14, 21 and 28, for comparison purposes. The rats were sacrificed after the 3-week training program, and the infarct volume was obtained using the triphenyltetrazolium chloride (TTC) method.

### Middle cerebral artery occlusion (MCAo)

The induction procedure of an MCAo surgery was based on Longa’s brain endovascular occlusion method^27^. Rats were anesthetized with intraperitoneal injection of Ketamine (50 mg/kg; Nankuang Pharmaceutical, Tainan, Taiwan), Atropine (0.03 mg/kg; Sintong Chemical, Taoyuan City, Taiwan) and Xylazine (10 mg/kg; Bayer AG, Leverkusen, Germany) cocktail. If required and depending on a reflex withdrawal response and breathing rate, an additional dose of the KAX cocktail (i.p.) was administered to maintain anesthesia. Body core temperature was thermostatically maintained at 37 °C with a feedback-controlled heating pad (PhysioSuite, Kent Scientific, Torrington, CT, USA) during surgery. After the rats were anesthetized, a midline incision was performed at the neck to expose the left common carotid artery (CCA), the external carotid artery (ECA) and the internal carotid artery (ICA). A white suture was used to tie the left CCA and the ECA. Then, a 4-0 monofilament nylon suture (Doccol Corporation, Redlands, CA, USA) was inserted into the ECA and then the ICA, until the middle cerebral artery (MCA) was blocked to induce a cerebral ischemic stroke. Sixty minutes later, reperfusion was achieved by withdrawal of the suture. The sham group only received operations involving ECA and ICA isolation without ligation. To relieve pain and discomfort in the postoperative period, topical 2% Lidocaine gel (Astra-Zeneca Pharmaceuticals, Wilmington, DE, USA) was applied on the wound, and Carprofen (5 mg/kg; YUNGSHIN PHARM IND. CO. LTD. Taichung, Taiwan) was injected subcutaneously for postoperative analgesia immediately after surgery and then daily, until the fifth postoperative days.

Successful ischemia during MCA occlusion was confirmed by reduction in cerebral blood flow (CBF) down to 20% of the original level using an OxyLite/OxyFlo (Oxford Optronics Ltd., Oxford, UK) fiber-optic laser Doppler system. Exclusion criteria were as stated follows: insufficient MCAo (a reduction in CBF to >20% of the baseline value), rats that died before the end of MCAo surgery, and without induction of brain ischemia (infarction) as quantified by TTC stain. Behavioral tests, as will be detailed below, were conducted as motor function assessments on all the animals by two investigators who were blinded to the experimental groups.

### Behavioral tests

For baseline performance and testing, animals were acclimatized to a testing room and experimental devices. One week before surgery, rats were placed, and freely walked on an inclined plane and a beam. Three days before surgery, rats were pre-trained to grab the inclined plane and traverse, but not fall off, the beam. If any of them failed the pre-training, it was excused before the group assignment.

#### Modified neurological severity score

The modified neurologic severity scores (mNSSs)^28^ is commonly used to assess the motor, sensory, reflex and balance function of testees, and it was conducted here on each testee before and after MCAO surgery. A testee scored a point in case it failed a task, mNSS was consequently rated on a scale of 0 to 18, and a more severe neural impairment is indicated by a higher mNSS. Baseline readings on Day −1 were used as the internal controls. All the behavioral experiments were performed between 10 AM and 3 PM.

#### Inclined plane test

An inclined plane test is a quantitative, objective and high sensitivity approach to assessing the gripping strength of the hindlimbs of rats after ischemic stroke^29^. The evaluation was primarily conducted to test the lasting grip strength of the forelimbs or hindlimbs in rats on a weekly basis. The inclined plane used in this study was constructed of a 60 cm × 60 cm incline adjustable acrylic panel. A motor and a ball screw were used to control the inclined angle of the acrylic plane from 0 (horizontal) to 70 degrees^29^. A rectangular box was placed on the acrylic plane to initially hold a rat, and a layer of Velcro was attached to the bottom of the box to allow the rat to grip with its fore or hindlimbs. This experimental process involved placing the rats in the rectangular box with their hind legs on the Velcro and fore legs on the acrylic plane. The inclined plane was slowly raised starting at 25 degrees, and the angle of the inclined plane was increased in 5-degree increments to determine the maximal angle at which a rat could hold onto the Velcro. At this point, the angle of the plane immediately stopped rising, and the angle of inclination was recorded accordingly.

#### Beam balance test

Motor coordination was evaluated in a quantitative manner using a beam balance test. The apparatus was as simple as the surface of an elevated wooden beam ((100 cm (L) × 5 cm (W) × 2 cm (H)) at a height of 10 cm from the ground. The number of times that a testee fell off the beam was recorded, and the performance was rated on a scale of 0 to 6. As tabulated in Table 1^30^, zero point represents “balances with steady posture and successfully reaching the goal box”, while 6 points represents fall off within a 20 s duration. Three trials were performed on each testee. The testees took a 10-min break in their home cages between trails. The final beam balance score was defined as the mean of the scores received in the three trials. In short, the higher the score, the poorer the balance performance.

**Table 1:** Beam balance test graded on a scale of 0 to 6 (normal = 0; maximum = 6).

Score	Behavior
0 point	Balances with steady posture and successfully reaching the goal box
1 point	Grasps side of beam
2 points	Hugs the beam and one limb falls down from the beam
3 points	Hugs the beam and two limbs fall down from the beam or spins on beam (>60 s)
4 points	Attempts to balance on the beam but falls off (>40 s)
5 points	Attempts to balance on the beam but falls off ( > 20 s)
6 points	Falls off: No attempt to balance or hang on to the beam (<20 s)

### Assessing cerebral infarction

The determination of brain infarction by triphenyltetrazolium chloride (TTC) staining is commonly used in rats with a stroke^13^. Four weeks after the MCAo, all of the rats were sacrificed, and their brains were carefully removed. A brain slicer was used to cut the brain coronal sections at a width of 2 mm from the top front brain. The fresh brain slices were incubated in 2,3,5-TTC at 37 °C for 30 min. The TTC-stained slices were observed under a microscope to determine the infarct volume caused by ischemic stroke in the rats. Normal cells appeared red after staining, whereas necrotic cells appeared white. To minimize the error introduced by edema and liquefaction after infarction, a corrected percentage of infarct volume^31^ was achieved using the following equation:

$$\begin{array}{c}{\rm{C}}{\rm{o}}{\rm{r}}{\rm{r}}{\rm{e}}{\rm{c}}{\rm{t}}{\rm{e}}{\rm{d}}\,{\rm{p}}{\rm{e}}{\rm{r}}{\rm{c}}{\rm{e}}{\rm{n}}{\rm{t}}{\rm{a}}{\rm{g}}{\rm{e}}\,{\rm{o}}{\rm{f}}\,{\rm{i}}{\rm{n}}{\rm{f}}{\rm{a}}{\rm{r}}{\rm{c}}{\rm{t}}\,{\rm{v}}{\rm{o}}{\rm{l}}{\rm{u}}{\rm{m}}{\rm{e}}\,({\rm{C}}{\rm{I}}{\rm{V}}{\rm{ \% }})\,=\,\{({\rm{c}}{\rm{o}}{\rm{n}}{\rm{t}}{\rm{r}}{\rm{a}}{\rm{l}}{\rm{a}}{\rm{t}}{\rm{e}}{\rm{r}}{\rm{a}}{\rm{l}}\,{\rm{h}}{\rm{e}}{\rm{m}}{\rm{i}}{\rm{s}}{\rm{p}}{\rm{h}}{\rm{e}}{\rm{r}}{\rm{i}}{\rm{c}}\,{\rm{v}}{\rm{o}}{\rm{l}}{\rm{u}}{\rm{m}}{\rm{e}}\\ -{\rm{i}}{\rm{p}}{\rm{s}}{\rm{i}}{\rm{l}}{\rm{a}}{\rm{t}}{\rm{e}}{\rm{r}}{\rm{a}}{\rm{l}}\,{\rm{n}}{\rm{o}}{\rm{n}}{\rm{i}}{\rm{n}}{\rm{f}}{\rm{a}}{\rm{r}}{\rm{c}}{\rm{t}}{\rm{e}}{\rm{d}}\,{\rm{v}}{\rm{o}}{\rm{l}}{\rm{u}}{\rm{m}}{\rm{e}})/{\rm{c}}{\rm{o}}{\rm{n}}{\rm{t}}{\rm{r}}{\rm{a}}{\rm{l}}{\rm{a}}{\rm{t}}{\rm{e}}{\rm{r}}{\rm{a}}{\rm{l}}\,{\rm{h}}{\rm{e}}{\rm{m}}{\rm{i}}{\rm{s}}{\rm{p}}{\rm{h}}{\rm{e}}{\rm{r}}{\rm{i}}{\rm{c}}\,{\rm{v}}{\rm{o}}{\rm{l}}{\rm{u}}{\rm{m}}{\rm{e}}\}\times 100.\end{array}$$

### Statistical analysis

All values are presented as mean ± standard error of mean (SD). Statistical analyses were performed using SigmaPlot 11.0 (Systat Software Inc., Chicago, IL, USA). To test the treatment effect on each of the behavioral tests, we used the repeated measures analysis of variance (ANOVA) followed by Mann-Whitney U comparisons. For infarction volume was assess by ANOVA followed by Fisher’s least significant difference (LSD) post-hoc test. If there was a significant difference, a Student’s t-test was used to compare variables for two groups. P values less than 0.05 was considered to be statistically significant.

### Ethics approval and consent to participate

The statements on Ethics approval and consent to participate in the study are reported in the Methods–Experimental animals section.

## Results

### Motor function

**Figure 7:**
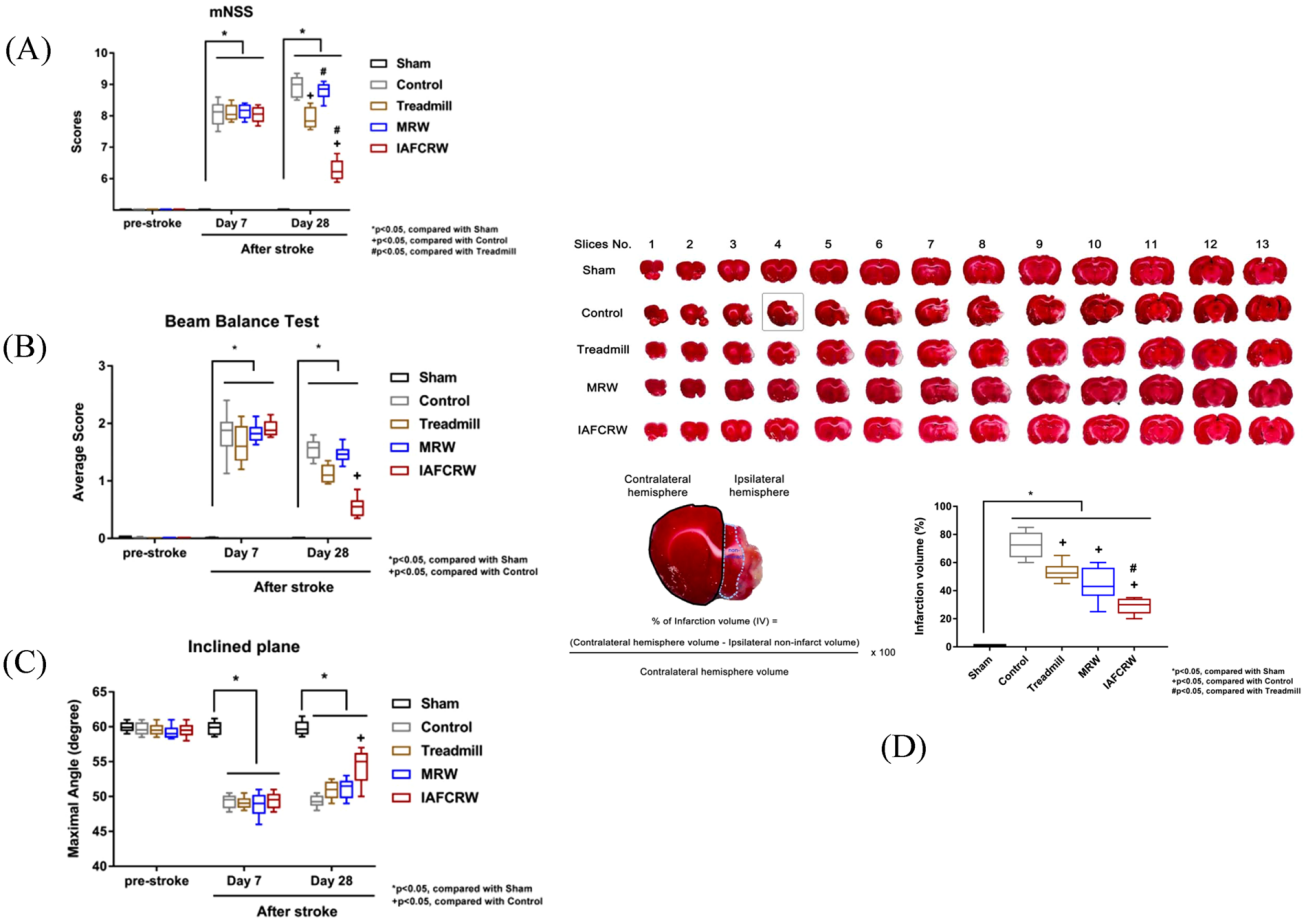
Rehabilitation performance comparison among groups. The neurological motor function was evaluated using (**A**) a mNSS, (**B**) a beam balance test, and (**C**) an inclined plane test before and after MCAo or sham surgery. All the rats displayed normal motor behavior at baseline (pre-stroke). On day 7 after stroke, but before rehabilitation, MCAo induction groups (including the control, treadmill, MRW and IAFCRW groups) of rats exhibited motor deficit behaviors, indicating that rats received successful stroke. (**D**) Representative TTC staining of 13 consecutive coronal brain sections of each group on day 28 after sham or MCAo is shown as an upper panel. TTC-stained 1 mm coronal brain slices show the white infarct area, and red color indicates intact tissue. The black line indicates the contralateral hemisphere area, while the light blue dotted lines indicate the ipsilateral non-infarct area. Infarcted volumes determined in the rats are shown as the percentage of the hemisphere. Data are expressed as mean ± SD of independent experiments (n = 10 for each group). *p < 0.05, compared with the sham group. ^#^p < 0.05, compared with the control group. ^+^p < 0.05, compared with the treadmill group. ^&^p < 0.05, compared with the MRW group.

Behavior test is known to be used to determine the severity of neurological motor dysfunction in rats after a stroke or MCAo. A higher average score in either the mNSS or a beam walking test, or a smaller inclination angle in an inclined plane test, indicates a more severe ischemic stroke injury. As illustrated in Fig. 7(A,B), the IAFCRW group was found to outperform the rest of the lesioned groups in terms of the average score received in the mNSS and the beam walking test over the 28-day period after injury. As illustrated in Fig. 7(C), the sham group gives an inclination angle of 60 ± 0.5 degrees, the highest among groups and a figure far beyond 50 ± 0.5 degrees in the control group. Moreover, the IAFCRW group received an angle of 56 ± 0.25 degrees on day 28, and significantly outperformed the treadmill and MRW groups. More importantly, the IAFCRW group was the only exercise group showing a significant recovery via stroke rehabilitation on day 28 after stroke. No significant difference was observed among the other two exercise groups (treadmill (52 ± 0.5 degrees) and MRW (51 ± 0.5 degrees)) and the control group.

### Infarct volume

TTC staining was then used to assess the infarct volume. Figure 7(D) presents a statistical comparison of the volume of dead brain cells among groups. As illustrated in Fig. 7(C), the infarct volumes in the treadmill (278 ± 18 mm^3^) and MRW (269 ± 22 mm^3^) groups were comparable to that in the control group (302 ± 15 mm^3^), while the IAFCRW group gave a significantly lower cerebral infarct volume (198 ± 12 mm^3^) than the control group (P < 0.05).

### Chance of reaching the threshold

**Figure 8:**
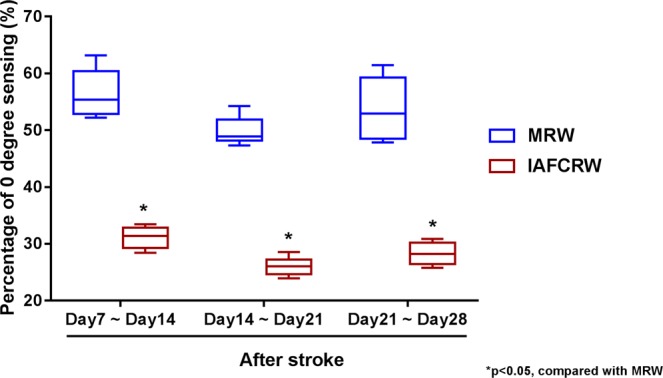
Comparison on the chance of reaching the threshold on a weekly basis between the IAFCRW and the MRW groups. The comparison results of IAFCRW vs. MRW were 32 ± 1% vs. 57 ± 3.5% during week 1, 26 ± 0.5% vs. 50 ± 2.5% during week 2, and 29 ± 1.5% vs. 54 ± 4.5% during week 3.

Rats with a stroke are known to have inferior motor functions and response, meaning that once they are brought up to the 0-degree position, they are very liable to cross the threshold, enter the unsafe zone, and fall consequently. Figure 8 gives respective chances of reaching the threshold in the IAFCRW and MRW groups on a weekly basis during the 3-week rehabilitation program. The comparison results of IAFCRW vs. MRW were 32 ± 1% vs. 57 ± 3.5% during week 1, 26 ± 0.5% vs. 50 ± 2.5% during week 2, and 29 ± 1.5% vs. 54 ± 4.5% during week 3. The average chance comparison over three weeks was 29 ± 1% vs. 53 ± 3.5%. As illustrated in Fig. 8, MRW gave chances above 50%, much higher than those in IAFCRW, simply due to the fact that the IAFCRW platform is physically a larger wheel than the MRW counterpart, and was designed as a rat-friendly rehabilitation environment.

## Discussion

In this study, it was found that rats with a surgically triggered stroke (such as MCAo) cannot be well rehabilitated at a fixed intensity, using existing rehabilitation platforms, since the rats’ motor functions were dulled and reduced. The experimental results showed that the IAFCRW group presented significant improvements in both the motor function recovery and the cerebral infarct volume with the control group as a benchmark (Fig. 7; P < 0.05) on Day 28, validating the performance of the IAFCRW platform. In contrast, there is an indistinguishable statistical difference in all the behavioral test results among the rehabilitated groups and the benchmark on Day 7, validating the argument that it takes time to observe the efficacy of rehabilitation.) Besides, significant differences in the motor function and the cerebral infarct volume were not observed as well among the treadmill, the MRW and the control groups, in agreement with two pieces of prior studies^13,14^. Hence, commercial forced exercise platforms were validated not as a well-designed stroke rehabilitation environment, and the outperformance of the IAFCRW platform was demonstrated accordingly.

Obviously, the IAFCRW platform has a great advantage over existing commercially available training platforms due to the use of the presented rehabilitation model. Even though there was a (29 ± $1$)% chance of reaching the threshold, the chance of fall was completely ruled out for the following reason. Once unable to keep pace with the running wheel, a rat was brought up to the threshold. Meanwhile, a deceleration mechanism was enabled instantly, the rat was brought back to the safe zone consequently, and then an acceleration mechanism was enabled to speed up the running wheel. In simple terms, the acceleration and deceleration mechanisms were enabled alternatingly, such that rats could be rehabilitated all the time in an uninterrupted, adaptive and fall-free manner, and resulted in the great outperformance relative to the MRW counterpart. It is worth noting that a significant efficacy of stroke rehabilitation was not observed in the MRW group, due to an up to (53 ± 3.5)% chance of reaching the threshold and the fact that MRW was not equipped with a deceleration mechanism. In other words, the MRW training was frequently interrupted by a fall over approximately half the training duration, and the trainee was made physically weaker each time after getting a fall injury. This partially accounts for the poor efficacy of rehabilitation in the MRW group. However, it must be pointed out that an IAFCRW group member, when reaching the threshold, did cling to a local vertical surface completely by limb strength, or it might slide down the runway right away. At this moment, the trainee can be seen as taking training at full capacity. As referred to previously, there were a (29 ± $1$)% chance of reaching the threshold and a zero chance of fall, meaning that the IAFCRW group members can be safely rehabilitated at full capacity for approximately one third of a training duration. This accounts for the outperformance of the IAFCRW platform in another sense.

The psychological stress interference, caused by electric shock when using treadmills, remains a key issue in the field of physiology, and might interrupt rehabilitation exercise. In this work, the treadmill group members were observed to be frequently electrically stimulated at the end of the treadmill, and were even to exhausted to be trained in the second half of a training duration, which is a major cause of second injury. Today’s commercially available MRWs cause as well training interference due to fall injuries. Therefore, the fall-free rehabilitation mechanism was developed herein in such a way that rats can be rehabilitated under a low level of stress interference, and analysis on collected raw neurophysiological data can be made more convincing by clinical researchers.

The training speed and the duration of a conventional training platform needs to be preset, and the wheel accelerates from rest to a specified training speed directly. However, this move makes it difficult for the rats to adapt to the running speed, and may cause injury, thereby deteriorating the rehabilitation performance. The presented adaptive acceleration model was developed based on the diverse physical characteristics of rats, and the rehabilitated members were permitted to gradually adapt to the final training speed. An advantage of this design is an improved repeatability of a training process. Another clear advantage is the feature to record all the running states and monitor the position of a trainee throughout the entire training process, a feature not available in commercially available platforms. A fixed training intensity and a duration are employed to estimate the total amount of exercise in commercial platforms, which may involve invalid or ineffective exercises, such as electric shocks, abnormal running, falls, etc. Recording of all the running states and position monitoring may help the physiological laboratory personnel understand the overall training condition of animals, and may as well account for the variability of experimental results. As a consequence, a deeper understanding and more convincing arguments can be made using the presented mechanism.

Table 2 presents a feature and hardware cost comparison among platforms. In addition to the above-referred advantages, the IAFCRW platform has a hardware cost advantage over counterparts. The hardware cost therein refers to the expense of building a platform, but excluding those of medical treatments. The fees for medical treatments, e.g. MCAo surgeries and rehabilitation expense, are exactly the same among various training platforms, meaning that there is no need to compare such medical treatment costs.

**Table 2:** Feature and hardware cost comparison among platforms for cerebral stroke rehabilitation purposes.

Features	Treadmill^13^	Motorized running wheel^14^	IAFCRW (this study)
Driving mode	Motorized and electrically stimulated	Centrally motorized	Laterally motorized
running speed	Fixed	Fixed	Variable
Speed control mechanism	Not available	Not available	Feedback adjusted
Number of Rats trained at a time	Multiple	Single	Single
Automated acceleration training	Not available	Not available	Available
Automated deceleration training	Not available	Not available	Available
Track material	Rubber belt	Cross bar	Acrylic and rubber belt
Threshold sensing	Not available	Not available	Available
Effective exercise assessment	Not available	Not available	Available
Fall prevention mechanism	Not available	Not available	Available
Training interference level	High	Intermediate	Low
Hardware cost	High	Intermediate	Lower-Intermediate

## Conclusion

This study presented a fall-free, adaptive rehabilitation mechanism for ischemic stroke rat patients, which was implemented in an IAFCRW platform. The severity of an ischemic stroke caused by MCAo in rats can be evaluated using 3 behavioral tests. The IAFCRW platform was found to outperform a traditional running wheel counterpart in terms of motor function and infarct volume, and to provide a completely safe and adaptive rehabilitation environment according to individual differences. The issue of motion rehabilitation had been explored for disease treatment in neurophysiology and pathology. However, most existing forced animal training platforms inevitably produced interfering factors, including shocks and falls, which were presumed to be reduced to a great extent herein. A significant recovery (Fig. 7; P < 0.05) was achieved in motor function tests and cerebral infarct volume measurements. Moreover, the IAFCRW group was the only exercise group showing a significant recovery through stroke rehabilitation, and the group members thereof can be safely rehabilitated all the time, and particularly at full capacity for approximately one third of a training duration. Therefore, this platform can be used by clinical researchers as a training platform for experimental verification purposes in future studies.

## Data Availability

The data that support the findings of this study are available from the corresponding author upon reasonable request.
